# RAAS in diabetic retinopathy: mechanisms and therapies

**DOI:** 10.20945/2359-4292-2023-0292

**Published:** 2024-04-22

**Authors:** Xin Li, Yu-Hong Fu, Xue-Wei Tong, Yi-Tong Zhang, Yong-Yan Shan, Yu-Xin Xu, Sheng-Dan Pu, Xin-Yuan Gao

**Affiliations:** 1 First Affiliated Hospital of Harbin Medical University Harbin Medical University Department of Endocrinology Harbin China First Affiliated Hospital of Harbin Medical University, Harbin Medical University, Department of Endocrinology, Harbin, China

**Keywords:** Diabetic retinopathy, renin-angiotensin-aldosterone system, renin, prorenin

## Abstract

Diabetic retinopathy (DR) is a complication of diabetes with a complex pathophysiology and multiple factors involved. Recently, it has been found that the upregulation of the renin-angiotensin-aldosterone system (RAAS) leads to overexpression of angiotensin II (Ang II), which induces oxidative stress, inflammation, and angiogenesis in the retina. Therefore, RAAS may be a promising therapeutic target in DR. Notably, RAAS inhibitors are often used in the treatment of hypertension. Still, the potential role and mechanism of DR must be further studied. In this review, we discuss and summarize the pathology and potential therapeutic goals of RAAS in DR.

## INTRODUCTION

Diabetic retinopathy (DR) is a potentially blinding ocular disease that is frequently observed in patients with long-term diabetes mellitus (1). The renin-aldosterone system (RAAS), one of the oldest studied hormone systems in the body, is well known for its roles in systemic vascular control and electrolyte homeostasis (2). One of the few tissues with local RAAS secretion is the retina, in which local production is suggested by a concentration of angiotensin II (Ang II) exceeding that of the circulation (2). The role of Ang II in the normal retina is most likely that of retinal homeostasis, including blood vessel constriction, regulation of glial cell function, and modulation of neuronal function (3). It is well established that all RAAS components are expressed in retinal cells (4), including (pro)renin, renin, angiotensinogen, angiotensin I (Ang I), Ang II, angiotensin-(1-7) (Ang-[1-7]), angiotensin-converting enzyme (ACE), ACE type 2 (ACE2), (pro)renin receptor ([P]RR), angiotensin II type 1 receptors (AT_1_R), angiotensin II type 2 receptors (AT_2_R), and Mas receptor (5–7). The discovery of ocular RAAS components provides evidence that RAAS is probably involved in the occurrence and development of DR. A Canadian study of longevity in patients with type 1 diabetes (T1DM) has also shown that RAAS activation is associated with DR (8). Therefore, RAAS may be a promising therapeutic target in DR treatment.

In this review, we aim to discuss the role of RAAS and the beneficial effects of its inhibitors in the progression of DR.

## THE ROLE OF RAAS IN OCULAR PHYSIOLOGY

### Local and circulating RAAS

**Figure 1:**
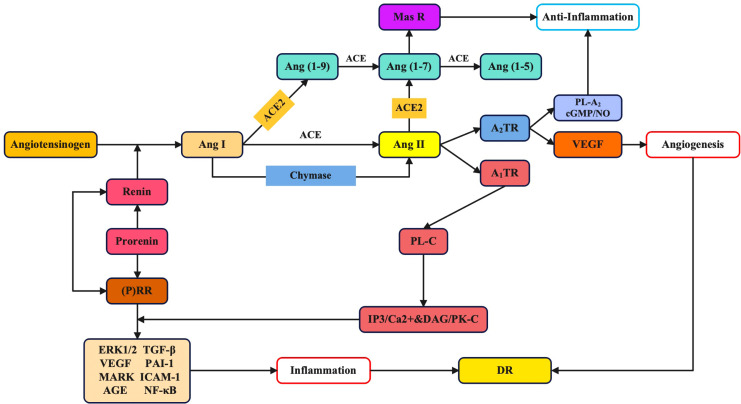
Involvement of the renin-angiotensin-aldosterone system in biochemical pathways and molecules in diabetic retinopathy.

Abbreviations: DR, diabetic retinopathy; RAAS, renin-angiotensin-aldosterone system; Ang I, angiotensin I, Ang II, angiotensin II; ACE, angiotensin-converting enzyme; ACE2, angiotensin-converting enzyme type 2; (P)RR, (pro)renin receptor; AT1R, angiotensin II type 1 receptors; AT2R, angiotensin II type 2 receptors; ERK 1/2, extracellular signal-regulated kinase 1/2; MAPK, mitogen-activated protein kinase; VEGF, vascular endothelial growth factor; AGE, advanced glycation end product; TGF-β, transforming growth factor-β; ICAM-1, intracellular adhesion molecule-1; NF-κB, nuclear factor-kappa B; PAI-1, plasminogen activator inhibitor-1; DAG/PK-C, diacylglycerol/protein kinase-C; IP3, inositol-1,4,5-triphosphate; NO, nitric oxide; PL-A2, phospholipase A2; PL-C, phospholipase C; cGMP, cyclic guanosine monophosphate.

Prorenin is activated to form renin in the juxtaglomerular cells of the kidney (Figure 1). Renin then binds to liver-produced angiotensinogen to generate the decapeptide Ang I, which is then hydrolyzed by ACE present in circulation or locally within tissues to produce the oligopeptide Ang II (9). Additionally, Ang II adjusts multiple physiological effects by activating AT_1_R and AT_2_R to transmit signaling. In the retina, Ang II mainly elicits pathologic effects through AT_1_R (10), whereas the actions of AT_2_R may be regulated differently from those of AT_1_R and, possibly, be contrary to the vasodilatory effects of AT_1_R (the effects of AT_2_R remain unclear). Previous studies suggested that circulating RAAS components (such as Ang I, Ang II, and angiotensinogen in plasma) were unable to enter ocular tissues. However, after the discovery of renin mRNA in the eyes, the hypothesis that ocular RAAS components are synthesized locally was confirmed (11).

Renin expression in retinal pigment epithelium (RPE) has been shown to be decreased in mice after systemic administration of Ang II. Additionally, systemic administration of enalapril, an ACE inhibitor (ACEI), increases renin expression levels in the retina and kidney in mice; specifically, enalapril increased 20 times the renin expression level in RPE. The systemic infusion of losartan, an AT_1_R blocker, can prevent the Ang II-dependent downregulated expression of renin in RPE (12). These findings suggest that circulating RAAS may regulate ocular RAAS. Thus, local RAAS components may play a vital role in ocular diseases.

### The three major RAAS signaling pathways

Notably, Ang II is mostly catalyzed by the enzyme ACE (*i.e.*, the classical pathway), although it can also be catalyzed by other enzymes, including chymase, a gastrointestinal enzyme also expressed in ocular tissues (13,14). The signal transmission of Ang II has three pathways, including the ACE/Ang II/AT_1_R axis and chymase/Ang II axis, the ACE2/Ang-(1-7)/Mas axis, and the prorenin and (P)RR pathway.

#### ACE/Ang II/AT_1_R axis and chymase/Ang II axis

The ACE/Ang II/AT_1_R axis is known as the classical signaling pathway. The RPE cells express AT_1_R, transient-receptor-potential channel-V2 (TRPV2) and angiotensin-receptor-associated protein (Atrap). Specifically, AT_1_R and Atrap are located at the basolateral membrane of the RPE (15); AT_1_R is a Gq protein-associated G-protein coupled receptor, and Ang II bonds with AT_1_R to activate diacylglycerol-protein kinase C (DAG/PK-C) and inositol-1,4,5-triphosphate (IP3)/Ca^2+^ signaling cascades (16). The signaling cascades increase intracellular Ca^2+^ levels due to the transient release of Ca^2+^ via IP3 receptor and TRPV2 channels in the endoplasmic reticulum. In ocular diseases like DR and age-related macular degeneration (AMD), the signaling cascades enhance the accumulation of inflammatory and angiogenic cytokines, including extracellular signal-regulated kinase (ERK) (17), mitogen-activated protein kinase (MAPK) (18), vascular endothelial growth factor (VEGF) (19), intracellular adhesion molecule-1 (ICAM-1) (20), nuclear factor kappa B (NF-κB), transforming growth factor-β1 (TGF-β1) (21), reactive oxygen species (ROS) (22), advanced glycation end products (AGEs) (23), and nicotinamide adenine dinucleotide phosphate (NADP[H]). These cytokines disrupt cell growth and intracellular signaling. Thus, these findings support the hypothesis that the ACE/Ang II /AT_1_R axis regulates ocular pathophysiology through inflammation.

In addition, chymase can also induce the expression of Ang II from angiotensin-(1-12) (Ang-[1-12]), which is independent of the ACE pathway (24). Chymase can induce oxidative stress via chymase-enhanced Ang II to attack the pancreas in diabetic animal models. Furthermore, by inhibiting chymase, the production of Ang II can be reduced (25). Therefore, chymase may be a potential target in preventing DR progression.

#### ACE2/Ang-(1-7)/Mas axis

Notably, ACE2 – a homolog of ACE – has also been described in the human retina (26). It can transform Ang II into Ang-(1-7), which acts contrarily to Ang II through a novel angiotensin receptor (Mas). The Mas receptor is a G-protein coupled receptor that induces telangiectasis and antiproliferative, antiinflammatory, and antifibrotic actions and is active in body fluid homeostasis (27). This type of receptor has been found in the retina and ciliary body. The ACE2/Ang-(1-7)/Mas axis is considered a protective axis that offsets the negative cardiac and vascular effects induced by activation of the ACE/Ang II/AT_1_R axis (28). Additionally, the Mas receptor and AT2R have been found to be interdependent in stimulating nitric oxide (NO) (29).

The activation of ACE2 mitigates lipopolysaccharide-induced inflammatory action in human RPE cells (30). The Otsuka Long-Evans Tokushima Fatty (OLETF) rat with diabetes mellitus is an animal model for examination of the expression of amyloid β peptide (Aβ) in the diabetic retina. In the OLETF rat retina, the accumulation of Aβ peptide is enhanced by high plasma glucose concentration (31). In human RPE cells, the inflammation activated by Aβ can be improved via activation of ACE2/Ang-(1-7)/Mas axis (32). The increased expression of ACE2 modulates the local immune responses to lessen ocular inflammation in mice with experimental autoimmune uveitis. The effects are mediated by activating the Ang-(1-7)/Mas pathways and inhibiting the NF-κB, signal transducer and activator of transcription 3 (STAT3), and MAPK signaling pathways (33). Additionally, ACE2 has been proven to exert beneficial effects in mice with diabetes by upregulating adeno-associated virus (AAV)-mediated gene delivery (34). Intraocular administration of AAV-ACE2/Ang-(1-7) to diabetic rats and mice significantly reduces diabetic retinal vascular leakage, acellular capillaries, leukocyte infiltration, and oxidative damage. Therefore, experimental data suggest that RAAS overexpression in the eye is associated with DR.

#### Prorenin and (pro)renin receptor pathway

Apart from AT_1_R, (P)RR also plays an important physiological role in blood pressure regulation and cellular function, including inflammation, proliferation, angiogenesis, and activation of growth factors (35). Prorenin, the precursor of renin, circulates at high concentrations in plasma and was considered previously to be physiologically inert. Levels of prorenin are high in persons with diabetes. The high level of prorenin is now used as a biomarker to predict diabetic microvascular complications.

Prorenin and its receptor play a significant part in the RAAS because their binding is a rate-limiting step. Prorenin binds to the (P)RR and undergoes conformational structural change, exposing its active site and exhibiting enzymatic activity (36). The binding is not the conventional prorenin proteolysis, which triggers a signaling transduction pathway independent of Ang II (37). This is called a receptor-associated prorenin system (RAPS), which plays a significant part in DR (38). The binding of prorenin and (P)RR increases the formation of angiotensin in specific tissues and transduces signaling by the classic RAAS pathway. The nonproteolytic activation of prorenin selectively accelerates pathologic retinal neovascularization via inflammatory processes (39). Prorenin also increases the generation of Ang II by enhancing renin activity. In addition, the binding of prorenin and (P)RR also directly stimulates MAPKs of (P)RR, including phosphorylation of ERK 1/2 (40). Studies in mesangial cells indicate that inhibition of (P)RR may play an important role in preventing DR independent from Ang II blockade (41).

Handle region peptide (HRP), a peptide derived from the prosegment of prorenin, is a (P)RR inhibitor that has shown beneficial effects in ocular pathophysiology by suppressing ERK activation and generation of vascular endothelial growth factor (VEGF) in AT_1_R-deficient diabetic mice (42). In addition, independent of the progression of DR, (P)RR involves the production of VEGF and its tyrosine kinase receptors. Further, (P)RR initiates the expression of angiogenic cytokines, such as ERK1/2, TGF-β1, and VEGF/VEGF receptor-2 in the retina, which can be blocked via (P)RR/ERK signaling (43,44). Thus, prorenin and (P)RR, which take part in the upstream of RAAS to regulate physiological function, can be novel therapy targets in DR.

All in all, RAAS is an integral part of the physical function of the retina. All the possible signaling pathways discussed above play significant roles in regulating ocular physiology. The local RAAS function may be controlled by RAAS inhibitors, including renin inhibitors, AT_1_R blockers (AT^1^RBs), ACEI, and (P)RR blockers. The RAAS components affect the physiological function of the retina. The components also play a role in the progression of DR. These provide a promising therapeutic strategy in the pathologic progress of DR.

## HYPERGLYCEMIA-INDUCES RAAS UPREGULATION

Hyperglycemia causes the upregulation of major glucose metabolic pathways, including the tricarboxylic acid cycle and glycolysis, as compensation for diabetic conditions. This compensatory mechanism increases the tricarboxylic acid cycle and leads to excessive accumulation of the intermediate succinate. The G protein-coupled receptor 91 (GPR91), also known as the succinate receptor, is expressed in the retinal ganglion cell layer. In retinal ganglion cells, succinate accumulates remarkably under ischemic conditions. GPR91 is activated via ERK1/2/cyclooxygenase-2 (COX-2)/prostaglandin E2 (PGE2) pathways to mediate VEGF-induced retinal vascular alteration. In DR, succinate activates GPR91 to induce VEGF expression (45). In addition, high glucose levels induce a paracrine mechanism involving GPR91 and succinate. Succinate binds to GPR91, initiating intracellular signaling transduction, including the release of intracellular Ca^2+^, activation of PGE2, and generation of NO. It directly activates the synthesis and release of renin (46). Hence, hyperglycemia increases renin release via the accumulation of succinate and GPR91 signaling pathways.

Nε-carboxymethyl lysine (CML) is a typical advanced glycation end product (AGE) that is formed as the result of the glycoxidation reaction of serine, lysine, and glucose (47). Hyperglycemia triggers the intracellular generation of CML. Further, CML induces NF-κB/p38/MAPK-dependent signaling transduction pathways, activating inducible nitric oxide synthase (iNOS) and, later, elevating NO formation (46).

Studies suggest that hyperglycemia elevates the amount of (P)RR in the plasma membrane of renal collecting duct cells. In M-1 cells treated with high glucose, (P)RRs are mainly localized at the surface of the plasma membrane, while in control rats, (P)RRs are located intracellularly. The augmented bind of (P)RR and prorenin increases the generation of renin (48). The hyperglycemia-induced increased (P)RR in the plasma membrane may be a novel mechanism to clarify the progression of DR. In conclusion, hyperglycemia activates RAAS overexpression in various ways, including through AGEs, GPR91, and (P)RR.

## RAAS INVOLVEMENT IN DIABETIC RETINOPATHY

The pathophysiological mechanisms of DR involve various complex factors. The upregulation of RAAS, including overexpressed Ang II, induces alterations of multiple pathways in the progression of DR, such as oxidative stress, inflammation, and vascular proliferation. Moreover, aldosterone may play a role in DR, and the pathological pathways are interconnected. The detailed mechanisms are discussed below.

### RAAS augments vascular proliferation and permeability

The RAAS upregulates the angiogenic cytokine VEGF probably via the Ang II/AT_1_R axis, leading to angiogenesis. In cultured mesenchymal stem cells, VEGF mRNA and VEGF protein levels increase after Ang II administration. The enhanced synthesis of VEGF has been speculated to occur via AT_1_R signaling transduction in ERK1/2 and protein kinase B (Akt) pathways (49). Further, Ang II-mediated VEGF induces angiogenesis (50) and increases retinal vascular permeability (51), increasing the possibility of hyperpermeability and neovascularization. Retinal neovascularization converts nonproliferative DR into proliferative DR, which expedites the DR progression. The blood-retinal barrier (BRB) is essential for a normal physiological function of the retinal microenvironment and low permeability (52). The BRB breakdown plays a critical role in the early pathogenesis of DR. In studies, the loss of tight junction proteins is induced by Ang II-mediated VEGF overexpression. Meanwhile, perindopril, an ACE inhibitor, can prevent the loss of tight junction proteins by blocking VEGFs. The recovery of the loss reduces the enhanced diabetic retina vessel permeability (53). Thus, RAAS increases angiogenesis and permeability through VEGF overexpression.

Apart from increasing angiogenesis, Ang II also plays a role in vessel remodeling. Pericyte loss is a major part of DR progression. In capillaries, pericyte cells regulate vessel tone and perfusion pressure, much like smooth muscle cells in larger vessels. The loss of pericytes decreases inner retina stability, making the inner retina vulnerable to hyperglycemia-induced pathological damage. In addition, the loss of pericyte triggers retinal vascular remodeling and retinal vasculature structure damage, results in abnormal revascularization, and further promotes the occurrence of DR (54). It has been reported that Ang II induces the apoptosis of pericyte cells via intracellular signaling of integrin α_3_ and β_1_ (55). In mouse retina, pericyte apoptosis is induced by increased Ang II under high glucose via integrin. By blocking integrin α_3_ and β_1_, the pericyte loss induced by Ang II is decreased. Hence, RAAS affects the revascularization of the retina via pericyte loss. Taken together, it seems that RAAS signaling pathways contribute heavily to pathological vessel events in DR.

### RAAS induces oxidative stress

Notably, Ang II is one of the main components inducing oxidative stress and producing ROS. Further, ROS is well known in the progression of DR. In cultured retinal ganglion cells, AT_1_RB signaling is involved in oxidative stress-induced retinal neurodegeneration (56). This is considered a major pathophysiological event in DR. Additionally, NADP(H) oxidase is induced by Ang II, which proves the occurrence of a connection between RAAS and ROS. It is well known that AT_1_R signaling activates protein kinase C (PKC), especially its alpha isoform, which induces vascular NADP(H) oxidase to overexpress the superoxide radical (57). In addition, Ang II also mediates the overexpression of leukotriene B_4_ (LTB_4_), leading to NADP(H) oxidase activation (58). Activated NADP(H)-oxidase is necessary for diabetes-induced retinal leukostasis (59). Blockades of AT1R effectively block diabetes-induced inflammatory response and oxidative stress including AGEs (60), ICAM-1, NF-κB, and NADP(H) oxidase (61), and increase neuroprotective agents (22). Besides, in the human retinal pigment epithelial cell line-19 (ARPE-19), hyperglycemia increases VEGF expression and activation in an NADP(H) oxidase mechanism via prorenin receptor, which is independent of Ang II (62). Thus, oxidative stress can be induced by RAAS in both Ang II and prorenin pathways.

The apoptosis of retinal capillary pericytes is known as a crucial event in the breakdown of the inner BRB, which is a major step in the progression of DR. Further, NADP(H) oxidase-induced ROS produce much more damage than mitochondrial dysfunction-derived ROS under hyperglycemia condition in triggering caspase-3-induced apoptosis of retinal capillary pericytes. It has been proven that intracellular CML-modified proteins are produced by NADP(H) oxidase-derived ROS rather than mitochondria-derived ROS (63). Hence, the interrelationship of RAAS and NADP(H) oxidase-derived ROS is also connected with AGEs. The RPE cells are a crucial part of the outer BRB and are susceptible to hyperglycemia and ROS concentration (64). Therefore, Ang II enhances the activation of NADP(H) oxidase-induced ROS production, which may play a significant part in the pathogenesis of DR.

### RAAS induces proinflammation in endothelial cells

Changes in inflammatory molecules have been detected in both diabetic animals and human retinas. These changes lead to increased BRB permeability and ischemia, driving angiogenesis (65). Further, Ang II activates NF-κB, and activated NF-κB induces the transcription of inflammatory mediators, including cellular adhesion molecules and VEGF (53). Increased levels of intracellular adhesion molecules indicate the occurrence of leukocyte recruitment and adhesion to the endothelium, and the subsequent fluid extravasation could damage the BRB. In animal models, increased Ang II concentration causes leukocytosis in the diabetic retina, which seems to be induced via overexpression of VEGF (66). The interaction of leukocytes and endothelial cells plays a significant part in the early stage of DR. Leukocytosis is a crucial step in endothelial cell dysfunction, so it may be possible that Ang II is involved in endothelial dysfunction.

Among adhesion molecules, ICAM-1 plays the most effective role in Ang II-induced retinal leukocytosis (67). Meanwhile, in the NF-κB signaling pathway, tumor necrosis factor-α (TNF-α) and interleukins are the most efficient proinflammatory mediators in retinal inflammation induced by Ang II. Interleukin-1β (IL-1β) also activates NF-κB, which increases the loss of pericytes in diabetic mouse retina (68). Therefore, Ang II-induced inflammation of endothelial cells leads to the breakdown of BRB in DR progression.

### The role of aldosterone and endothelin-2 in ocular diseases

Aldosterone is a mineralocorticoid synthesized in the zona glomerulosa of the adrenal cortex (69). The enzyme that regulates aldosterone production is aldosterone synthase (cytochrome P450 family 11, subfamily B, member 2), which regulates gene expression by potassium and Ang II (70). Aldosterone plays an important role in maintaining sodium and water in the body by acting on mineralocorticoid receptors (MRs) in the distal renal tubules (71). Several studies have found that aldosterone synthase, 11β-hydroxysteroid dehydrogenase type 2 (11β-HSD2), and MRs are expressed in Müller cells, retinal microvascular cells, and retinal ganglion cells (72,73). The MRs expressed in Müller cells are implicated in DR pathology.

Endothelin-2, a family member of potent vasoconstrictors and a possible mediator of Müller cells in monitoring photoreceptor damages (74), has been reported to be increased in serum in a mouse model of DR (75). Recent studies have found that inhibition of AT_1_R or MR reduces vascular leakage induced by intravitreal injection of endothelin-2 in mice, suggesting an association between RAAS and Müller cell dysfunction induced by endothelin-2 (76). Moreover, Ohashi and cols. examined the effects of high glucose conditions on MR in human retinal Müller cells *in vitro*. High-glucose treatment induces Müller cell swelling with increased expression of MR protein, which is suppressed by an MR antagonist (eplerenone) (77). A study by Zhao and cols. reported that MR is overexpressed in the retina of type 2 diabetes (T2DM) Goto-Kakizaki rats and humans (78); thus, local MR antagonism could be a novel therapeutic option for DR.

## THE EFFECTIVE AND POTENTIAL THERAPEUTIC TARGETS OF RAAS

### Clinical studies with ACEI/angiotensin II receptor blockers

Notably, ACEI and Ang II receptor blockers (ARBs) have shown effects on inhibiting DR progression. Preclinical studies have shown that ACEI and ARBs reduce retinal vascular leakage and VEGF levels as well as degeneration of retinal capillaries in diabetes (23,79). Both ACEI and ARBs have been shown to protect DR by reducing the overexpression of ocular VEGF/VEGF receptor-2 (80,81). Additionally, ARBs are relevant to the ACE2/Ang-(1-7)/Mas axis and can enhance the expressions of ACE2 and Ang-(1-7)/Mas and downregulate the expression of inflammatory cytokines, including interferon-c (IFN-c), interleukin-6 (IL-6), IL-1β, and TNF-α. Olmesartan, an ARB, blocks AGE-induced vascular cell adhesion molecule-1 (VCAM-1) gene expression by restoring the downregulated levels of ACE2 and stimulating the Ang-(1-7)/Mas receptor axis in renal mesangial cells (82). Telmisartan, an ACEI, may upregulate the ACE2/Ang-(1-7)/Mas axis to prevent apoptosis of retinal vascular endothelial cells (83).

Moreover, it has been found that AT1RBs can also recover the downregulation of glyoxalase-1 (GLO-1), a key regulator of AGE information, which is mediated by Ang II in retinal vascular cells (23). Retinal ganglion cell loss occurs in optic nerve inflammation. In an animal model of multiple sclerosis highly associated with retinal ganglion cell loss, the concentration of Ang II increases at an early stage, and after administration of candesartan, the retinal ganglion cell loss is reduced (84). Further, ACEI is effective in decreasing optic nerve inflammation. A meta-analysis of the effects of RAAS inhibitors in diabetic retinopathy has indicated that RAAS inhibitors could reduce the incidence and progression of DR and suggested that ACEI offers many more benefits than ARBs in DR (85). Despite effective results, RAAS is not blocked, due to a compensating feedback mechanism (86). Additionally, ACEI in low doses could not significantly slow down the progression of DR in patients with T2DM who had normal blood pressure, suggesting that the ACEI dose has an effect on ocular RAAS blockade (87). Thus, the conventional view that Ang II is a crucial therapeutic target must be reconsidered.

The Diabetic Retinopathy Candesartan Trials (DIRECT) failed to show benefits of candesartan, an ACEI, in preventing DR progression in patients with T1DM, while in those with T2DM, candesartan treatment resulted in a 34% regression of DR (88). Subsequent findings from DIRECT indicated that the presence of microaneurysms predicted an increased risk of DR progression in individuals with T1DM and T2DM, while ARB reduced the risk of microaneurysm progression (89). These results suggest a beneficial effect of candesartan in DR. Furthermore, a multicenter trial published by Mauer and cols. has found that retinopathy progression was reduced by 65% with enalapril (an ACE inhibitor) and by 70% with losartan (an AT_1_R blocker) (90). These findings confirm that AT_1_R blockade provides benefits for individuals with DR.

### Clinical studies of renin and (P)RR

Direct renin inhibitors (DRIs), such as aliskiren, are also a potential treatment for DR and have the advantage of acting directly on renin compared with traditional RAAS inhibitors. In fact, DRIs are a limiting step in the activation of the signaling cytokine cascade and may be more effective in blocking RAAS. Wilkinson-Berka and cols. have shown that aliskiren offers similar or better protective effects on the retina in models of retinal disease (91). Additionally, aliskiren inhibits RAAS in RPE cells exposed to aliskiren (92). Endothelial progenitor cells (EPCs) are involved in the process of angiogenesis via VEGF and chemokine stromal cell-derived factor-1a (SDF-1a) (93), which can contribute to the repair of injured vessels (94). High glucose levels reduce EPC function (95), which may lead to the development of vascular complications in diabetes (96,97). Aliskiren improves the function of human EPCs in hyperglycemia status, which is related to upregulated VEGF/SDF-1a (98). This evidence supports the hypothesis that the upstream components of RAAS could be better targets in inhibiting RAAS function, although the mechanisms remain unclear. These findings indicate a novel and promising therapeutic target in preventing the development of DR.

### Clinical studies of aldosterone/MR

The MR is an intracellular steroid hormone receptor and a member of the nuclear receptor superfamily of proteins. The development of the steroidal MR antagonists spironolactone and eplerenone led to convincing evidence from preclinical studies that MR blockade reduces tissue damage in various diseases, especially chronic kidney disease (99). Finerenone is a novel nonsteroidal MR antagonist. Results of the trials Finerenone in Reducing Kidney Failure and Disease Progression in Diabetic Kidney Disease (FIDELIO-DKD) and Finerenone in Reducing Cardiovascular Mortality and Morbidity in Diabetic Kidney Disease (FIGARO-DKD) have shown that finerenone significantly reduces kidney and cardiovascular outcomes (100,101), indicating that finerenone might have therapeutic benefit in other diabetic complications such as DR (102). A study by Jerome and cols. (103) reported that finerenone has beneficial effects on the retina of rodents with diabetes and ischemic retinopathy, reducing the hallmark features of vision-threatening vascular injury that includes a breakdown of the BRB and neovascularization, as well as a reduction in retinal inflammation. These findings suggest that finerenone might be a potential new oral treatment for patients with DR. The findings are consistent with a prior study in Goto-Kakizaki rats with T2DM that demonstrated that the intraocular administration of spironolactone reduced retinal vascular leakage and edema, although retinal VEGF levels were not reduced (78).

### Clinical studies of the new pathway

Meanwhile, VEGF mediates the breakdown of the BRB to cause diabetic macula edema (DME) and promotes pre-retinal neovascularization, which leads to vitreous hemorrhage, tractional retinal detachment, and neovascular glaucoma (104). Therefore, intravitreal injection of anti-VEGF agents has become the standard treatment to improve visual acuity in DME (105). However, DME refractory to anti-VEGF treatment is reported to have a prevalence of approximately 40% in landmark clinical trials (106). A study using human retinal endothelial cells has shown that RAAS activation reduces the efficacy of anti-VEGF in reclosing the barrier, indicating that RAAS inhibitors may be a potential treatment modality for refractory DME (107).

Exploration of the angiopoietin (ANG) tyrosine kinase endothelial receptor (Tie) pathway has shown encouraging results for the management of AMD and DME (108). In adults, the ANG/Tie pathway is involved in the regulation of vascular homeostasis, modulation of vascular permeability, and neoangiogenic and proinflammatory processes (109). In detail, Tie-2 is a transmembrane receptor selectively located on endothelial cells in blood vessels and acts as a binding site for ANG1 and ANG2 (110). These latter molecules exert a different biological activity; ANG1 is a full Tie-2 agonist, inducing its phosphorylation and the activation of a downstream signaling process, which causes a positive effect on vascular stability and inhibits vessel permeability and leakage; in contrast, ANG2 acts as a partial agonist/antagonist of Tie-2. Therefore, its binding to the Tie-2 receptor prevents the activation of the entire pathway and leads to vascular leakage (111). Interestingly, preclinical studies have highlighted that the simultaneous dual inhibition of ANG2 and VEGF-A is superior to inhibition of VEGF-A or ANG2 alone (112).

Faricimab is the first bispecific antibody designed for intraocular use. Its antigen-binding fragments independently inhibit ANG2 and VEGF-A with high affinity and specificity. YOSEMITE (NCT03622580) and RHINE (NCT03622593) were two multicenter, randomized, double-masked, phase III clinical studies of DEM demonstrating that robust vision gains and anatomical improvements with faricimab were achieved with adjustable dosing up to every 16 weeks (113). These findings suggest that faricimab may be a potential new treatment for patients with DR.

In conclusion, overall, the retinal RAAS component has attracted the attention of scholars. Ocular RAAS has been proven to contribute to augmenting permeability, enhancing cell proliferation, and inducing proinflammation and oxidative stress during the development of DR. The inhibition of RAAS has shown some effects in clinical trials. Besides, the chymase-induced Ang II pathway is an independent endocrine pathway that could be a target for new therapeutic methods. The DRI and MR antagonists could also be new therapeutic targets in preventing the progression of DR. Meanwhile, more research about therapies for RAAS in DR is necessary, and so are more clinical trials to confirm the benefits and safety of RAAS inhibitors in DR. Therefore, we look forward to relevant studies like these in the future.
